# The Health Status of the Reproductive System in Women Living In the Aral Sea Region

**DOI:** 10.3889/oamjms.2015.078

**Published:** 2015-06-23

**Authors:** Yasminur G. Turdybekova, Raushan S. Dosmagambetova, Symbat U. Zhanabayeva, Gena V. Bublik, Alik B. Kubayev, Zhanbolat G. Ibraibekov, Irina L. Kopobayeva, Berikbay Zh. Kultanov

**Affiliations:** Karaganda State Medical University, Department of Obstetrics and Gynecology, Molecular Biology and Medical Genetics, Gogol 40, Karaganda 1000008, Kazakhstan

**Keywords:** women’s reproductive health, environmental factors, Aral Sea region, hormonal status, ecological crisis

## Abstract

In order to assess women’s reproductive health in the Kyzylorda region (the Aral Sea) of Kazakhstan, 1406 women were involved in an integrated clinical-functional and laboratory examination, given regional and environmental ecological factors. The high level of endocrine gynecological pathology is indicated in the examined women. In both examined zones, there is a late menarche over 16 years old, which is 39%. It is indicated a trend towards younger age of menopause onset. Inflammatory diseases of the female genital organs affect a third of the examined women. In the zone of ecological disaster, every fourth woman has fetal losses, cases of spontaneous pregnancy termination and/or non-developing pregnancies in anamnesis, which can be repeated many times.

## Introduction

Kyzylorda region problem has acquired threatening scale in 60s of the twentieth century. Intensive reclamation, further development of irrigated agriculture, construction of irrigation systems throughout Central Asia, continuing water demand growth of domestic and industrial consumption created conditions for one of the largest in recent history global environmental catastrophe – drying of ponds. Over the past fifty years, an area of the Aral Sea has shrunk by more than four times; volume has decreased by ten times, as well as its mineralization [1].

According to researchers it was established that the excess of norm of lead content in the soil and its maximum permissible concentration (MPC) in all of selected samples. Moreover, according least removal from factory concentration lead in soil decreased: in the first zone lead content in soil exceed MPC from 163 till 17.28 times; in the second zone - from 26.47 to 1.16 times; the third zone - from 9.06 to 3.28 times [2].

According to the center of sanitary-epidemiological expertise of the Republic of Kazakhstan in 2014, the content in the soil of the Aral region of heavy metals (Ra-226, Cs-137) and pesticides exceed maximum permissible concentration (MPC) and the maximum dust concentration reaches 2.4 MPC, nitrogen dioxide - 6.4 MPC, carbon oxide 2.2 MPC - practically, this is a real smog. The Syr Darya maximum level exceeded the norm for sulphate - 12.9 times, nitrites – 9.3 times, copper - 15 times, magnesium - 3.6 times. Average annual concentrations of pollutants exceeded the norm by 1.2 to 4.4 MPC. The maximum level of contamination had been seen in the spring period. Index of water pollution in the river Syr Darya was 1.18.

In Kazakhstan, negative trends of the environment reflected on health and demographic processes of the Kyzylorda region’s population. Choosing the basic criteria, the most objective is not only nature and extent of environmental pollution, but also dynamics of such health indicators as infant, maternal and total mortality and fertility, average life expectancy of the population. In recent years, in the Aral Sea region of Kazakhstan there is a birth rate increase from 22.7 (2004) to 28.45 (2013), mortality rate decrease to 6.6 (2004 – 7.2), increase in the rate of natural population growth to 21.79 (15.5 in 2004) per 1 000 population in 2013. Despite the positive changes in the demographic situation, a low level of women health and children remains. The maternal mortality rate in the dynamics of the last 5 years is being reduced, so in 2013 it was 10.4 per 100 000 born alive (68.4 – 2008). The main causes of maternal mortality are obstetric bleeding, gestosis, extragenital pathology, due to high levels of abortion and morbidity (infections, sexually transmitted infections, and anemia). Despite the downward trend in the infant mortality rate (from 23.89‰ in 2008 to 19.1‰ in 2013 to 1000 born alive), mortality in low birth weight and preterm infants was 60.3%. The main cause of infant mortality is perinatal pathology (66.3%), congenital malformations (14.4%) are in the second place, respiratory diseases (7.6%) – on the third [3, 4]. The aim of our research is an evaluation of the women’s reproductive health of the Aral Sea region in an integrated clinical-functional and laboratory examination, taking into account regional and environmental ecological factors.

## Material and Methods

Examination subject are territories of five localities of the Kyzylorda region. The selection to groups was implemented according the stratification principle (by gender) and quota equal selection of women for the following groups: 18-29 years old, 30-39, 40-49 in each locality. The inclusion criterion for examination was the factor of residence of reproductive age women (18-49 years old) in the area of ecological trouble for at least 5 years, employment in occupations with harmful conditions not higher than the second class. The examination material was blood and vaginal contents. Clinic-instrumental research: cervix examination in mirrors smears on purity degree, oncocitology, bimanual examination, breast examination, vaginal content pH determination.

## Results

A zone of ecological crisis is Zhalagash, Zhusaly, Shieli villages, where, respectively, 199, 173, 348 women of fertile age were examined. Total number of examined is 1406 women. In both examined areas, there is late menarche older than 16 years (39.5%). In the crisis zone, it is 41%, while in the ecological disaster zone is 38%. Late menarche is recorded significantly more likely than early one, it amounts 5.7% in urban and 10.5% in rural residents. At late menarche, for a long time, the right cyclical menstruation of the absolute majority of girls is not set, one-third of them have rare and/or scanty menstruation until fertile age and primary oligomenorrhea is diagnosed in such cases. Late menarche is most typical for girls underweight, with chronic disease, with increased mental and physical activity, with toxic effect on body.

In the gynecological morbidity analysis, it should be noted that 34% (every third woman) has inflammatory diseases of uterus and appendages. Inflammatory processes prevail in the age groups of 30-39 and 40-49 years old. From nosological forms, vulvovaginitis and bacterial vaginosis dominate in the age group of 18-29, salpingoophoritis – in 30-39. Coleitis, uterine fibroids, endometriosis are found most frequently in the women aged 40-49.

The largest number of women suffering from bacterial vaginosis was found in the age group of 30-39 years old that is 14.5%, 18-29 years old – 10.6%, 40-49 years old – 9.8%. The obtained results of smear examination with cytological signs ASCUS (atypical squamous cells of undetermined significance, including inflammation) were found in the age group of 18-29 years old – 3%, 30-39 years old – 5%, 40-49 years old – 4.3%. According to the literature data, most women are healthy, 11.6% has signs of CIN II and CIN III, and one among a thousand has cervical cancer [9].

According to our research, cytological picture of CIN I and CIN II dysplasia is most common for aged 40-49. For identified cases it is most common in Aiteke-Bi village, CIN I 2.5% and CIN II 1.7% aged 30 – 39, CIN I 7.7% and CIN II 6.4% aged 40-49. Tactics of these women conducting may differ from many factors. It is important to diagnose and treat inflammatory, dishormonal and other diseases of genitals, carry out correction of immunity if there are indications.

The presence of genitals inflammatory and background processes increases the risk of infertility, both female and male, complications of pregnancy and childbirth. The World Health Organization (WHO) estimates that the frequency of infertility in women account for 40%, in men – 45%, and 15% due to the presence of spouse incompatibility and immunological factors. According to our research, 3% of women with primary infertility and 2% with secondary infertility were found in Aiteke-bi village; 3% with primary and 5% with secondary infertility – in Aralsk village. In Zhalagash village, primary infertility was found in 3% and secondary - in 4% of women. In Zhusaly village, it was found 2% of women unable to give birth to first child, and 6% of women already having children but not able to give birth again. 3% of women with primary and 7% with secondary infertility were identified in Shieli village.

According to initial estimates of the WHO, 5% of the population is recognized as sterile by anatomical, genetic, endocrine and immunological reasons. There are about 48.5 million of infertile couples in the world. 19.2 million of them experience difficulties with first child birth. Problems with ovulation (36% of cases), fallopian tubes (30% of cases) and endometriosis (18%) are the major medical causes of infertility [5, 7].

Five women with premature menopause before 40 years old were identified in Aralsk. Early menopause was observed in 7 women aged from 40 to 45. In Aiteke-bi village, 10 premature and 7 early cases of menopause were observed. In Zhalagash village, it was revealed 1 case of premature menopause and 4 cases of early. In Zhusaly village, early menopause was identified only in 4 women; Shieli village – 4 cases of premature and 2 cases of early menopause. According to the WHO, today the incidence of this pathology is estimated from 1 to 3% of the female population to 10%. This condition is described as «a multifactorial syndrome, which development may be caused by genetic, immune and environmental factors». Two-thirds of women have involvement of immune mechanisms, autoimmune reactions. Autoantibodies can damage ovaries and other organs such as thyroid gland, activating apoptosis in their cells. Other possible causes are viral infections, chromosomal abnormalities (most often Turner syndrome or various X chromosome defects). Chemical toxins are considered as possible environmental factors [6, 8].

Perinatal losses in women in the ecological disaster zone of Kazakhstan are 24%, in the ecological crisis zone – 23%. Every fourth woman has a history of spontaneous abortion cases and/or non-developing pregnancy, which may be repeated many times. The abortions frequency increases with age: 15% in the group of 18-29 years old and 32% in the late reproductive age. In anamnesis, the case of spontaneous abortion was occurred once in 44%, two – 11%, three – 8%, four – 1%.

In the examined groups of reproductive age women, number of abortions was 18%. It should be noted that as age increases the number of women using abortion as a contraceptive method also increases. In the age group 18-29 years old, the abortion rate is 7%, 29-39 years old – 20%, late reproductive age – 28%.

According to the research, 34% of women use contraception methods. In the group aged 18-29, this figure was 27%. The most frequent contraceptive method used in this age group is a barrier method (57%), 23% – lactational amenorrhea method (LAM), 19% – intrauterine method, 1% - other methods. In the group aged 30-39, 42% of women use contraceptive methods. This age group is more likely to use intrauterine contraceptive method – 68%, LAM – 14%, barrier method – 15%, progestin contraceptives and combined oral contraceptives – 1%, sterilization performed on indications during cesarean section – 2%. Women aged 40-49 are practicing contraceptive method in 33% of cases. The most preferred method of contraception in this age group is intrauterine device – 76%, barrier method – 20%, sterilization – 4%. All examined reproductive aged women living in ecological disaster and environmental crisis zone of the Kyzylorda region were passed blood plasma laboratory examinations in order to assess the hormonal status. According to the examination results, multidirectional changes in prolactin and in testosterone hormone level were recorded in all age groups. The results are shown in Table 1.

**Table 1:** Indicators of hormonal analysis in the blood plasma of reproductive age women, (M ± m)

Regions/Indicators	Age years	Testosterone. nmol/l	Prolactin, mkme/l
Ecological disaster zone (Aiteke-bi village, Aralsk city)	18-29	4.9 ± 1.8	405.3 ± 101.26

	30-39	2.3 ± 0.34	388.36 ± 46.6

	40-49	2.45 ± 0.46	511 ± 33.3

Ecological crisis zone (Zhusaly village, Zhalagash village, Shieli village)	18-29	3.4 ± 0.68	408 ± 41.8

	30-39	3.6 ± 0.68	365.68 ± 98.12

	40-49	3.16 ± 0.91	390.22 ± 67.12

Note: - accuracy in comparison with the groups of different ecological zones, p <0.001

## Discussion

Thus, according to the obtained results of women’s reproductive health evaluation and clinical examination, it can be concluded that a high level of endocrine gynecological pathology is indicated. In both examined zones, there is a late menarche over 16 years old, which is 39%. It is indicated a trend towards younger age of menopause onset. Number of women with premature menopause before 40 years old prevails over early menopause by 9%. In the infertility structure, number of women with secondary infertility prevails. Perinatal losses in women in the ecological disaster zone are 24%. Every fourth woman has cases of spontaneous pregnancy termination and/or non-developing pregnancy in the anamnesis, which may be repeated many times. In this regard, we can assume that the effect of dust – salt aerosols high concentrations and high background radiation lead to an increase in the frequency of perinatal mortality among the examined women. Our assumption about the complex impact of negative environmental factors and toxicants on women’s reproductive health is consistent with the results of previous domestic and foreign scholar’s researches.
